# Rapid Purification of a New P-I Class Metalloproteinase from *Bothrops moojeni* Venom with Antiplatelet Activity

**DOI:** 10.1155/2014/352420

**Published:** 2014-06-01

**Authors:** Mayara R. de Queiroz, Carla C. Neves Mamede, Kelly C. Fonseca, Nadia C. G. de Morais, Bruna B. de Sousa, Norival A. Santos-Filho, Marcelo E. Beletti, Eliane C. Arantes, Leonilda Stanziola, Fábio de Oliveira

**Affiliations:** ^1^Instituto de Genética e Bioquímica, Universidade Federal de Uberlândia, 38400-902 Uberlândia, MG, Brazil; ^2^Instituto Nacional de Ciência e Tecnologia em Nano-Biofarmacêutica (N-Biofar), 31270-901 Belo Horizonte, MG, Brazil; ^3^Faculdade de Ciências Farmacêuticas de Ribeirão Preto, Universidade de São Paulo, 14040-903 Ribeirão Preto, SP, Brazil; ^4^Instituto de Ciências Biomédicas, Universidade Federal de Uberlândia, 38400-902 Uberlândia, MG, Brazil

## Abstract

The present study aimed to evaluate the proteolytic and biological activities of a new metalloproteinase from *B. moojeni* venom. The purification of BmooMP**α**-II was carried out through two chromatographic steps (ion-exchange and affinity). BmooMP**α**-II is a monomeric protein with an apparent molecular mass of 22.5 kDa on SDS-PAGE 14% under nonreducing conditions. The N-terminal sequence (FSPRYIELVVVADHGMFTKYKSNLN) revealed homology with other snake venom metalloproteinases, mainly among P-I class. BmooMP**α**-II cleaves A**α**-chain of fibrinogen followed by B**β**-chain, and does not show any effect on the **γ**-chain. Its optimum temperature and pH for the fibrinogenolytic activity were 30–50°C and pH 8, respectively. The inhibitory effects of EDTA and 1,10-phenantroline on the fibrinogenolytic activity suggest that BmooMP**α**-II is a metalloproteinase. This proteinase was devoid of haemorrhagic, coagulant, or anticoagulant activities. BmooMP**α**-II caused morphological alterations in liver, lung, kidney, and muscle of Swiss mice. The enzymatically active protein yet inhibited collagen, ADP, and ristocetin-induced platelet aggregation in a concentration-dependent manner. Our results suggest that BmooMP**α**-II contributes to the toxic effect of the envenomation and that more investigations to elucidate the mechanisms of inhibition of platelet aggregation may contribute to the studies of snake venom on thrombotic disorders.

## 1. Introduction

Snake venoms comprise a complex mixture of proteins, organic compounds with low molecular mass, and inorganic compounds [1–3]. The biologically active proteins and peptides induce a wide variety of effects on preys and human victims [4]. Viperid venoms contain powerful enzymatic and nonenzymatic components that affect haemostatic mechanism, such as proteinases, phospholipases A_2_, and disintegrins [5–14].

Snake Venom Metalloproteinases (SVMPs) play a key role in local tissue damage and systemic alterations resulting from viperid snake envenomations. These enzymes can induce haemorrhage, necrosis, oedema, skin damage, and inflammation, and degrade extracellular matrix components and impair the regeneration of affected skeletal muscle [6, 9, 15–17]. SVMPs can also affect platelet function through specific structural or enzymatic effects on platelet receptors or their ligands and the coagulation cascade by multiple mechanisms [2, 13, 15, 18].

In Brazil,* Bothrops* snakes are responsible for more than 90% of all snakebites in which the species was identified, representing a serious medical problem [19].* B. moojeni* is a snake that occurs in central and southeastern Brazil and adjacent countries including Paraguay and Argentina.* B. moojeni* are predominantly found in riparian vegetation in the central Brazilian savannahs, such as gallery forests and adjacent wet grasslands, although they are occasionally found in drier interfluvial areas [20]. This snake is responsible for the majority of snakebite accidents that occurred in the Triângulo Mineiro region and were registered in the Hospital of Clinics of the Federal University of Uberlândia-MG. In this work, we describe the purification, determination of N-terminal amino acid sequence, and functional characterisation of BmooMP*α*-II, a metalloproteinase from* B. moojeni* snake venom with antiplatelet activity.

## 2. Materials and Methods

### 2.1. Materials

Desiccated* B. moojeni* venom was purchased from Bioagents Serpentarium (Batatais, SP, Brazil). Acrylamide, ammonium bicarbonate, ammonium persulphate, aprotinin, benzamidine, bromophenol blue, ethylenediaminetetraacetic acid (EDTA), bovine fibrinogen, *β*-mercaptoethanol, leupeptin, N,N′-methylene-bis-acrylamide, sodium dodecyl sulphate (SDS), N,N,N′,N′-tetramethylethylenediamine (TEMED), 1,10-phenanthroline, and DEAE-Sephacel column were from Sigma Chemical Co. (St. Louis, MO, USA). Glycine, tris, molecular weight markers for electrophoresis, and benzamidine-sepharose column were purchased from GE Healthcare (Uppsala, Sweden). All the agonists used in the platelet aggregation assays (collagen, adenosine diphosphate and ristocetin) were purchased from Helena Laboratories (Beaumont, Texas, USA). All other reagents used were of analytical grade.

### 2.2. Animals

Swiss male mice (20–25 g) and Wistar male rats (200–250 g) were maintained under controlled temperature (22 ± 2°C), humidity (60–70%), and light/dark cycle (12 hours) with free access to food and water. The experiments were carried out in accordance with the current guidelines established by Ethical Committee in Animals Experimentation of Federal University of Uberlandia (Minas Gerais, Brazil; protocol 108/12).

### 2.3. Blood Collection

Human blood was obtained through donation from volunteers. The experiments reported here followed the guidelines established by the Human Research Ethics Committees of Universidade Federal de Uberlândia (CEP/UFU), Minas Gerais, Brazil (Protocol n° 055/11).

### 2.4. Isolation of BmooMP*α*-II

Crude venom of* B. moojeni* (400 mg) was dissolved in 50 mmol/L ammonium bicarbonate buffer, pH 7.8, and clarified by centrifugation at 10,000 ×g for 10 min. The supernatant solution was chromatographed on a DEAE-Sephacel column (2.5 × 20 cm) previously equilibrated with 50 mmol/L ammonium bicarbonate buffer, pH 7.8, and eluted with a concentration gradient (50 mmol/L–0.6 mol/L) of the same buffer. Elution was carried out at a flow rate of 20 mL/h and fractions of 3.0 mL/tube were collected. The fibrinogenolytic fraction (peak DS7) was pooled, lyophilised, and applied on a benzamidine-sepharose column, previously equilibrated with 50 mmol/L Tris-HCl and 500 mmol/L NaCl buffer (pH 7.4). The samples were eluted with 50 mmol/L glycine buffer, pH 3.0. Elution was carried out at a flow rate of 30 mL/h; fractions of 3.0 mL/tube were collected and their absorbances at 280 nm were read. The enzyme that was not absorbed by the column was named BmooMP*α*-II.

### 2.5. Estimation of Protein Concentration

Protein concentration was determined by the microbiuret method of Itzhaki and Gill [21], using bovine serum albumin as standard.

### 2.6. Electrophoretic Analysis

Polyacrylamide gel electrophoresis in the presence of sodium dodecyl sulphate (SDS-PAGE) was performed by the method of Laemmli [22] using 14% (w/v) gels. Electrophoresis was carried out at 20 mA/gel in Tris-glycine buffer, pH 8.3, containing 0.01% SDS. The molecular mass standard proteins used were phosphorylase b (97 kDa), bovine serum albumin (66 kDa), ovalbumin (45 kDa), carbonic anhydrase (30 kDa), soybean trypsin inhibitor (20.1 kDa), and *α*-lactalbumin (14.4 kDa). Gels were stained with Coomassie blue R-250, 0.2% (w/v). The relative molecular mass of BmooMP*α*-II was estimated by Kodak 1D image analysis software.

### 2.7. N-Terminal Sequencing

The N-terminal sequence of BmooMP*α*-II was determined by Edman degradation, performed on an automated sequenator model PPSQ-33A (Shimadzu Co., Kyoto, Japan). The identity of the primary sequence of BmooMP*α*-II compared with other proteins was evaluated using BLAST (http://blast.ncbi.nlm.nih.gov/Blast.cgi).

### 2.8. Proteolytic Activity upon Fibrinogen

Fibrinogenolytic activity was assayed as previously described by Rodrigues et al. [23], with modifications. Samples of 25 *μ*L of bovine fibrinogen (3 mg/mL saline) were incubated with 10 *μ*g of BmooMP*α*-II at 37°C for different periods of time (0, 15, 30, 60, and 120 minutes). The reaction was stopped with 0.0625 mol/L Tris-HCl buffer, pH 6.8, containing 10% (v/v) glycerol, 10% (v/v) *β*-mercaptoethanol, 0.2% (w/v) SDS, and 0.001% (w/v) bromophenol blue. Reaction products were analysed by 14% (w/v) SDS-PAGE. The effect of inhibitors on the fibrinogenolytic activity was assayed after preincubation of BmooMP*α*-II (10 *μ*g) in saline, pH 8.0, for 15 minutes with each of following inhibitors (5 mmol/L): EDTA, *β*-mercaptoethanol, 1,10-phenanthroline, benzamidine, leupeptin, and aprotinin. The thermal stability of BmooMP*α*-II was tested by preincubating the enzyme (10 *μ*g) in saline, pH 8.0, for 15 min at varying temperatures (30–90°C). Similarly, 10 *μ*g of BmooMP*α*-II was preincubated at different pH (4.0 to 10.0). The treated samples were added to bovine fibrinogen and incubated for 60 minutes at 37°C. The reaction was stopped and analysed by 14% (w/v) SDS-PAGE.

### 2.9. Haemorrhagic Activity

Haemorrhagic activity was determined by the method of Nikai et al. [24], with slight modifications. Test solutions of BmooMP*α*-II (100 *μ*g) were subcutaneously injected into the dorsal skin of mice (*n* = 3). Control animals received the same volume of sterile saline. After 3 hours, mice were sacrificed by overdose of ketamine/xylazine. The skin was removed and the diameter of haemorrhagic spot was measured on the inside surface.

### 2.10. Defibrinating Activity

Defibrinating activity was tested by the method of Gene et al. [25], with slight modifications. Groups of three Swiss male mice were injected i.p. with BmooMP*α*-II (100 *μ*g/100 *μ*L saline). Control animals received the same volume of sterile saline. After one hour, mice were sacrificed by overdose of ketamine/xylazine and bled by cardiac puncture. Whole blood was placed in tubes and kept at 25–30°C. Activity was determined by measuring the time until blood clotting onset.

### 2.11. Blood Clotting Activity

Clotting activity was assayed on platelet-rich plasma (PRP). Human blood collected in sodium citrate (3.2%) was centrifuged at 100 ×g for 12 minutes at room temperature to obtain PRP. BmooMP*α*-II (10 *μ*g/10 *μ*L saline) or the same volume of saline (negative control) or 0.2 mol/L calcium chloride (positive control) was added to 200 *μ*L of human PRP at 37°C. Clotting activity was determined by measuring the time until fibrin clot onset.

### 2.12. Histological Characterisation of Pathological Effects

Systemic histological alterations induced by BmooMP*α*-II in various organs were assayed as described by Costa et al. [6], with some modifications. Groups of four mice were injected i.p. with BmooMP*α*-II (50 *μ*g/100 *μ*L saline) or* B. moojeni* crude venom (50 *μ*g/100 *μ*L saline). Control animals received i.p. injection of 100 *μ*L of saline under identical conditions.

Myotoxic activity was assayed as described by Rodrigues et al. [17], with slight modifications. Groups of four mice were injected i.m. in the right gastrocnemius muscle with BmooMP*α*-II (50 *μ*g/50 *μ*L saline) or* B. moojeni* crude venom (50 *μ*g/50 *μ*L saline). Control animals received i.m. injection of 50 *μ*L of saline under identical conditions.

After 24 hours of injection, mice were sacrificed by overdose of ketamine/xylazine and heart, lung, liver, kidney, and right gastrocnemius muscle were dissected out. For histological analysis, the different tissues were then fixed in solution containing 10% (v/v) formalin, dehydrated by increasing concentrations of ethanol (70 a 100%), diaphanised with xylol, and embedded in paraffin. Thick sections (5 *μ*m) were cut in a microtome and stained with hematoxylin-eosin to be examined under a light microscope.

### 2.13. Platelet Aggregation

Platelet aggregation assays were performed in human PRP and measured using an automated 4 channel Aggregometer (AggRAMTM version 1.1, Helena Laboratories, USA). Human blood collected in sodium citrate (3.2%) was centrifuged at 100 ×g for 12 minutes at room temperature to obtain PRP. Platelet-poor plasma (PPP) was obtained from the residue by centrifugation of citrated blood at 1,000 ×g for 15 minutes. Assays were carried out using 200 *μ*L of PRP maintained at 37°C under continuous stirring in siliconized glass cuvettes. Aggregation was triggered with collagen (10 *μ*g/mL), ADP (20 *μ*M) or ristocetin (1.5 mg/mL) immediately after adding BmooMP*α*-II (20, 40 or 80 *μ*g) to human PRP. One hundred percent (100%) aggregation was expressed as the percentage absorbance relative to PPP aggregation. Control experiments were performed using only platelet agonists. All experiments were carried out in triplicate.

### 2.14. Statistical Analysis

The statistical analyses were carried out by ANOVA using the GraphPad prism program version 5.01. Differences with *P* values of less than 5% (*P* < 0.05) were considered significant.

## 3. Results and Discussion

**Figure 1:**
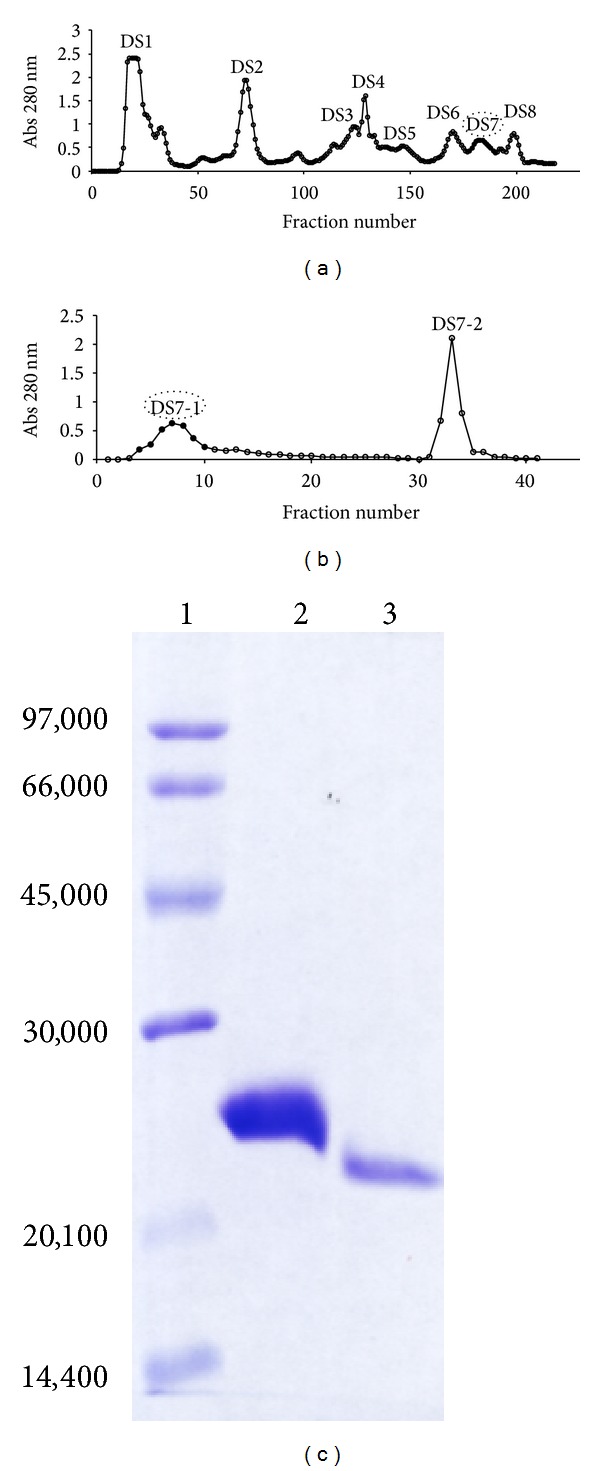
Purification of the BmooMP*α*-II from* B. moojeni* venom. (a) Separation on DEAE-Sephacel ion-exchange chromatography: crude venom (400 mg) was applied on the column (2.5 × 20 cm) and elution was carried out at a flow rate of 20 mL/h with ammonium bicarbonate gradients buffer (50 mmol/L–0.6 mol/L). Fractions of 3.0 mL/tube were collected and their absorbances at 280 nm were read. (b) Separation on benzamidine sepharose affinity chromatography: fraction DS7 was applied to the column previously equilibrated with 50 mmol/L Tris-HCl, 500 mmol/L NaCl, pH 7.4. After elution of the unbound fraction, 50 mmol/L glycine buffer, pH 3.0, was applied to the column and the absorbance of the fractions was monitored at 280 nm. Fractions of 3.0 mL/tube were collected at a flow rate of 30 mL/h. Pooled fractions are indicated by the closed circle. (c) SDS-PAGE in 14% (w/v) gel. Lanes: 1-standard proteins; 2-reduced BmooMP*α*-II; 3-non-reduced BmooMP*α*-II. The gel was stained with Coomassie blue R-250.

Proteolytic enzymes from snake venoms have attracted the interest of researchers due to their important role in envenomation caused by* Bothrops* snakes. In this work, we describe the purification and characterisation of a P-I SVMP from* B. moojeni* venom. The proteinase was purified from crude venom using a DEAE-Sephacel column producing eight main protein fractions (Figure 1(a)). The proteins present in the DS7 fraction showed substantial fibrinogenolytic activity (data not shown). The DS7 fraction was further fractioned using affinity chromatography on a benzamidine sepharose column (Figure 1(b)). The nonadsorbed fraction showed a single-band protein with great purity level, which we named BmooMP*α*-II. Electrophoretic analysis (SDS-PAGE) under reducing and nonreducing conditions indicated that the BmooMP*α*-II enzyme had a molecular mass about 25.5 and 22.5 kDa, respectively (Figure 1(c)). This molecular mass is similar to other bothropic SVMPs such as BjussuMP-II (24 kDa) from* B. jararacussu* [26], BmooMP*α*-I (24.5 kDa) from* B. moojeni* [27], Atroxlysin-I (23 kDa) from* B. atrox* [28], and BleucMP (23.5 kDa) from* B. leucurus* [10].

BmooMP*α*-II represents around 0.9% of the* B. moojeni* crude venom (data not shown). This yield was similar to the haemorrhagic metalloproteinase BlaH1 (0.8–1%) from* B. lanceolatus* venom [29]. In contrast, the yield was lower than several proteinases purified from bothropic venom by different methodologies such as BthMP [9] and BmooMP*α*-I [27] representing 2.3% and 8.7% of* B. moojeni* venom, respectively; BpSP-I represents about 3% of* B. pauloensis* venom [30] and Leucurobin represents about 3.5% of* B. leucurus* venom [31].

Both BmooMP*α*-II and BmooMP*α*-I (which was purified by Bernardes et al. [27]) were purified from the same snake venom. However, though both proteins are fibrinogenolytic enzymes and have similar molecular mass they differ from each other mainly because BmooMP*α*-II was obtained from the DS7 fraction while BmooMP*α*-I was obtained from the DS2 fraction.

**Figure 2:**
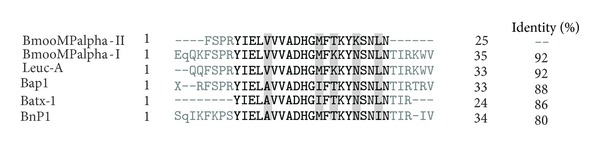
Sequence alignments of the initial N-terminal of BmooMP*α*-II with other metalloproteinases from P-I SVMP. BmooMP*α*-I from* B. moojeni* (see [27], P85314.2); leucurolysin-a from* B. leucurus* (see [32], P84907.2); Bap1 from* B. asper* (see [33–35], 1ND1_A); BaTX-I from* B. atrox* (see [36], P0DJE1.1); BnP1 from* Bothropoides pauloensis* (see [37], P0C6S0.1). The highly conserved residues are in bold, and those conserved are highlighted in gray.

BmooMP*α*-II was subjected to N-terminal sequencing by Edman degradation. The first 25 amino acid residues from N-terminal sequencing (FSPRYIELVVVADHGMFTKYKSNLN) were submitted to BLAST. The primary sequence of the BmooMP*α*-II shared a high degree of identity with other P-I SVMPs, such as BmooMP*α*-I from* B. moojeni* [27], leucurolysin-a from* B. leucurus* [32], Bap1 from* B. asper* [33–35], BaTX-I from* B. atrox* [36], and BnP1 from* Bothropoides pauloensis* [37] (Figure 2).

**Figure 3:**
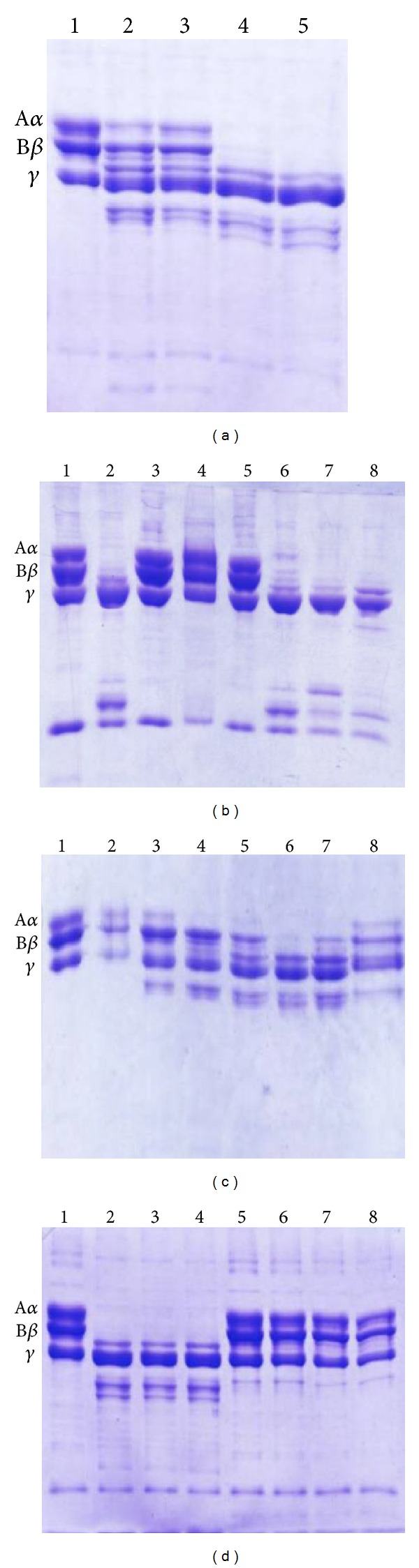
Proteolysis of bovine fibrinogen by BmooMP*α*-II. Line 1: negative control-fibrinogen incubated with enzyme for 0 minutes. (a) Proteolysis of bovine fibrinogen by BmooMP*α*-II time-dependent. Lanes 2–5: fibrinogen incubated with enzyme for 15, 30, 60 and 120 minutes, respectively. (b) Proteolysis of bovine fibrinogen by BmooMP*α*-II and effect of inhibitors. Lanes 2: positive control-fibrinogen incubated with enzyme for 60 minutes, 3–8: fibrinogen incubated with enzyme for 60 minutes after preincubation of BmooMP*α*-II with 5 mmol/L EDTA, 5 mmol/L *β*-mercaptoethanol, 5 mmol/L 1,10-phenanthroline, 5 mmol/L benzamidine, 5 mmol/L leupeptin, and 5 mmol/L aprotinin for 15 minutes, respectively. (c) Effect of the pH on the stability of BmooMP*α*-II. Lanes 2–8: fibrinogen incubated with enzyme for 60 minutes after preincubation of BmooMP*α*-II in pH 4.0, 5.0, 6.0, 7.0, 8.0, 9.0, and 10.0, respectively. (d) Effect of temperature on the stability of the BmooMP*α*-II. Lanes 2–8: fibrinogen incubated with enzyme for 60 minutes after preincubation of BmooMP*α*-II for 15 minutes at 30, 40, 50, 60, 70, 80, and 90°C, respectively.

Fibrinogenolytic enzymes found in snake venoms may be classified depending on the specificity of hydrolysis of the fibrinogen chains [2]. BmooMP*α*-II was active upon bovine fibrinogen, preferentially hydrolysing the A*α* chain followed by the B*β* chain, while apparently the *γ* chain was unchanged. A*α*- and B*β*-chain degradation was completed after 60 min incubation (Figure 3(a)). Therefore, BmooMP*α*-II is classified as *α*-fibrinogenase, similar to neuwiedase [23], BlaH1 [29], BjussuMP-II [26], BmooMP*α*-I [27], BthMP [9], and BleucMP [10].

BmooMP*α*-II is able to hydrolyze bovine fibrinogen by molecular mechanisms that are not understood. Okamoto et al. [38] showed that the BmooMP*α*-I is active upon neuro- and vasoactive peptides including angiotensin I, bradykinin, neurotensin, oxytocin, and substance P. Interestingly, BmooMP*α*-I showed a strong bias towards hydrolysis after proline residues, which is unusual for most of characterized peptidases. Moreover, BmooMP*α*-I showed kininogenase activity similar to that observed in plasma and cells by kallikrein [38].

BmooMP*α*-II fibrinogenolytic activity was completely inhibited by metal-chelating agents EDTA and 1,10-phenanthroline (Figure 3(b)), which remove metallic cofactors that are necessary for enzymatic catalysis [39]. These results, in addition to the sequence from the N-terminal and the molecular mass, corroborate the suggestion that this enzyme belongs to class P-I of the SVMPs. Moreover, the absence of haemorrhage caused by BmooMP*α*-II corroborates this finding. The fibrinogenolytic activity of the enzyme was also abolished by the reducing agent *β*-mercaptoethanol (Figure 3(b)), indicating that disulphide bonds are fundamental to the structural and functional integrity of BmooMP*α*-II. In addition, BmooMP*α*-II was not inhibited by benzamidine, leupeptin, or aprotinin (Figure 3(b)).

Stability tests upon bovine fibrinogen showed that the optimum pH for the proteolytic activity was pH 8.0, though the enzyme was partially active in the pH range of 5.0–10.0 (Figure 3(c)). Furthermore, BmooMP*α*-II showed proteolytic activity at temperatures of 30–50°C; however, at high temperatures (≥60°C), the fibrinogenolytic activity was fully lost (Figure 3(d)).

**Figure 4:**
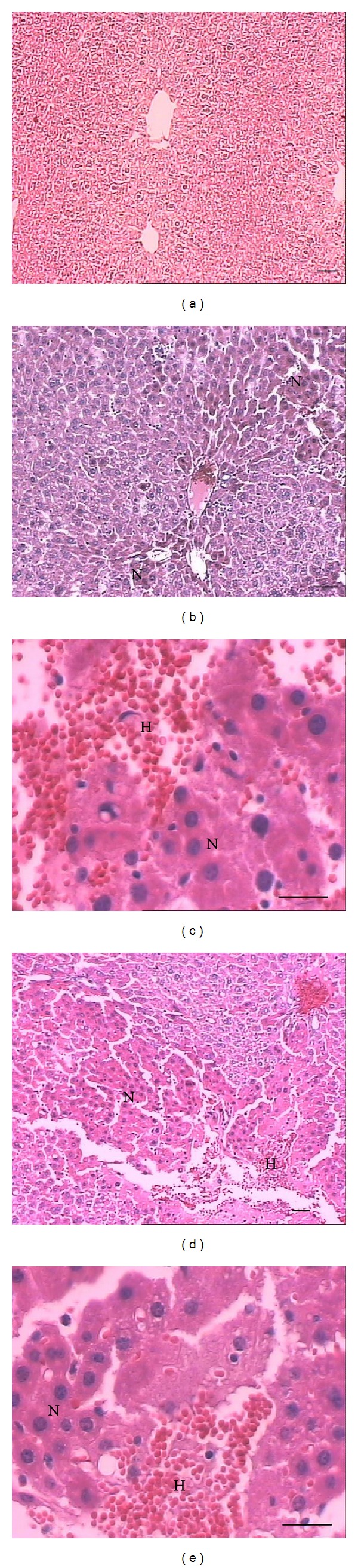
Light micrographs of section of mouse liver 24 hours after i.p. injection of* B. moojeni* crude venom or BmooMP*α*-II (50 *μ*g/100 *μ*L saline), stained with hematoxylineosin. (a) Control: normal tissue. (b-c) and (d-e)* B. moojeni* crude venom and BmooMP*α*-II, respectively: note necrosis and haemorrhage. Letters indicate the presence of necrosis (N) and haemorrhage (H). Scale bar = 100 *μ*m (a, b, d); scale bar = 50 *μ*m (c, e).

**Figure 5:**
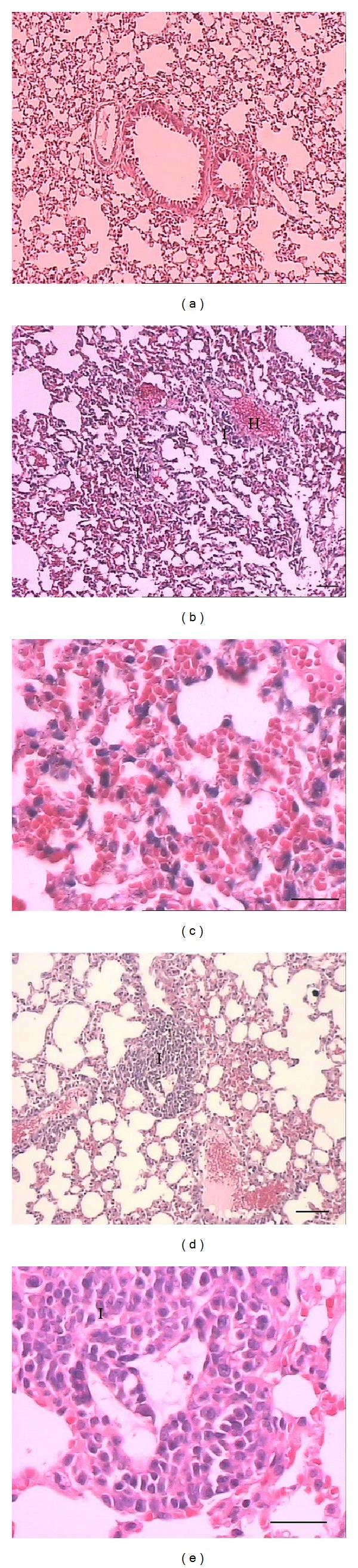
Light micrographs of section of mouse lung 24 hours after i.p. injection of* B. moojeni* crude venom or BmooMP*α*-II (50 *μ*g/100 *μ*L saline), stained with hematoxylin-eosin. (a) Control: normal tissue. (b-c) and (d-e)* B. moojeni* crude venom and BmooMP*α*-II, respectively: note pneumonitis evidenced by dilatation of the alveolar septa due to inflammatory infiltrate and hyperaemia. Letters indicate the presence of inflammatory infiltrate (I) and hyperaemia (H). Scale bar = 100 *μ*m (a, b, d); scale bar = 50 *μ*m (c, e).

**Figure 6:**
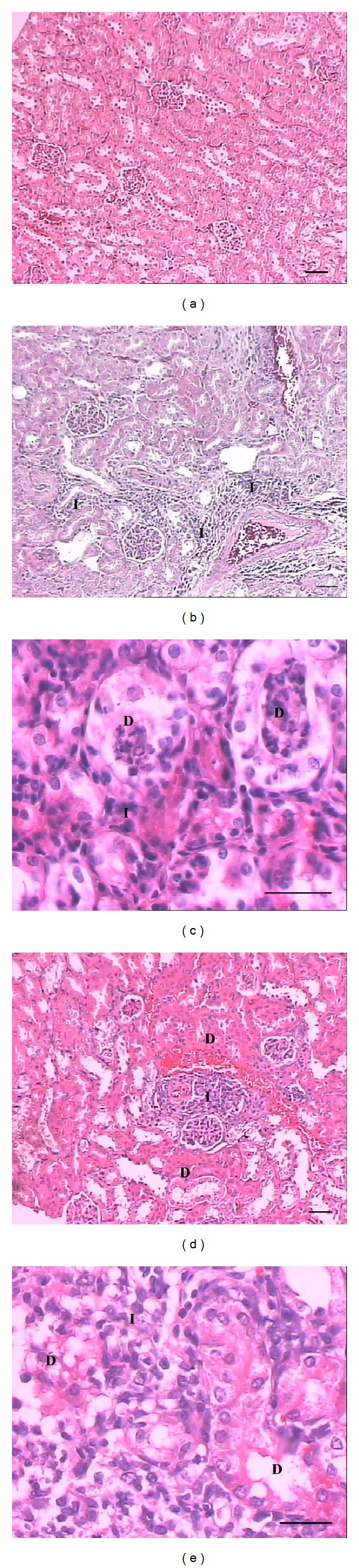
Light micrographs of section of mouse kidney 24 hours after i.p. injection of* B. moojeni* crude venom or BmooMP*α*-II (50 *μ*g/100 *μ*L saline), stained with hematoxylin-eosin. (a) Control: normal cortical region in the kidney. (b-c) and (d-e)* B. moojeni* crude venom and BmooMP*α*-II, respectively: note renal tubular degeneration with cell debris formation and inflammatory infiltrate. Letters indicate the presence of renal tubular degeneration (D) and inflammatory infiltrate (I). Scale bar = 100 *μ*m (a, b, d); scale bar = 50 *μ*m (c, e).

**Figure 7:**
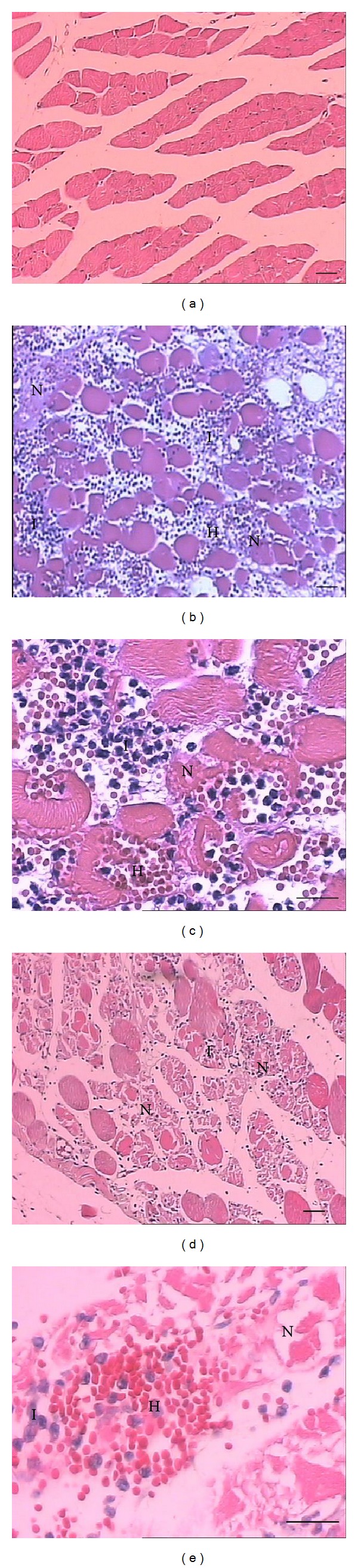
Histopathological analysis of myonecrosis induced by* B. moojeni* or BmooMP*α*-II. Light micrographs of sections of mouse gastrocnemius muscle 24 hours after i.m. injection of* B. moojeni* crude venom or BmooMP*α*-II (50 *μ*g/50 *μ*L saline), stained with hematoxylin-eosin. (a) Control: normal integer fibres are seen. (b-c) and (d-e)* B. moojeni* crude venom and BmooMP*α*-II, respectively: note myonecrosis evidenced by loss of cellular boundaries, cytoplasmic changes, no apparent striations and low or absent nuclei, accompanied by inflammatory infiltrate and haemorrhage. Letters indicate the presence of necrosis (N), inflammatory infiltrate (I) and haemorrhage (H). Scale bar = 100 *μ*m (a, b, d); Scale bar = 50 *μ*m (c, e).

Histological examination showed important morphological alterations in the liver, lung, kidney, and gastrocnemius muscle from mice caused by BmooMP*α*-II (Figures 4 to 7), which seem to contribute to the toxic effect of* B. moojeni* crude venom. In the heart, crude venom caused hyaline degeneration and haemolysis, while BmooMP*α*-II did not induce changes (results not shown). BmooMP*α*-II (Figures 4(d) and 4(e)) and crude venom (Figures 4(b) and 4(c)) induced haemorrhage in the liver evidenced by erythrocytes between the hepatocytes and not limited by the endothelium. Even in the liver, BmooMP*α*-II and venom caused necrosis characterised by loss of cellular boundaries, cytoplasmic changes, pyknosis, karyorrhexis, and karyolysis. In the lung, crude venom (Figures 5(b) and 5(c)) and BmooMP*α*-II (Figures 5(d) and 5(e)) caused pneumonitis evidenced by dilatation in the alveolar septa due to inflammatory infiltrate. Moreover,* B. moojeni* venom induced pulmonary hyperaemia.  Figure 6 shows that BmooMP*α*-II (Figures 6(d) and 6(e)) and* B. moojeni* crude venom (Figures 6(b) and 6(c)) induced renal tubule degeneration with formation of cell debris and inflammatory infiltrate. Direct nephrotoxic action of* B. moojeni* venom on tubule cells and glomerular structures is the most important physiopathologic factor in envenomation-induced renal failure [40]. Renal changes induced by BmooMP*α*-II can lead to renal failure.

Local tissue damage is a relevant problem caused by* Bothrops* snake envenomations, resulting from the combined action of several components of venom [6, 9, 12, 15, 41, 42]. BmooMP*α*-II and* B. moojeni* crude venom induced myonecrosis when compared with the control group. As expected, histological analysis of the control group showed the fibres intact, with clear striations and peripheral nuclei (Figure 7(a)). Therefore, the histological changes observed are due to action of inoculated toxins. BmooMP*α*-II (Figures 7(d) and 7(e)), as well as* B. moojeni* crude venom (Figures 7(b) and 7(c)), induced myonecrosis after 24 hours, evidenced by loss of cellular boundaries, cytoplasmic changes, no apparent striations, and few or absent nuclei, accompanied by inflammatory infiltrate and haemorrhage. Bleeding found in muscle tissue can be an indirect secondary effect of intense necrosis or due to the action of the metalloproteinase domain on vascular components after 24 hours of action* in vivo*. The mechanism of action of SVMP-induced muscle tissue damage is not well established. Some haemorrhagic SVMP-induced muscle damage is a secondary effect of ischaemia. However, others SVMPs can have a direct cytotoxic action on muscle cells or other unknown mechanisms [15].

Although a wide range of functional activities is assigned to SVMPs [3, 15], our results show that BmooMP*α*-II was unable to induce haemorrhagic, blood clotting, and defibrinating activities. Our results show that BmooMP*α*-II hydrolysed chains of the fibrinogen* in vitro* but did not cause defibrinating activity when administered i.p. to mice. This fact can be explained by inactivation of the enzyme by endogenous inhibitors of proteases present in the blood of animals.

The SVMPs have broad substrate specificity and have been shown to interfere by different mechanisms on platelet aggregation [13, 18]. The effects of several SVMPs on platelet aggregation are associated with the presence of disintegrin and disintegrin-like domains in P-II and P-III classes, respectively [5, 13, 43, 44]. Many P-II and P-III SVMPs that interfere on platelet aggregation have been purified and characterised [45–49]. However, there are few studies that show the action of PI SVMPs on inhibition of platelet aggregation.

**Figure 8:**
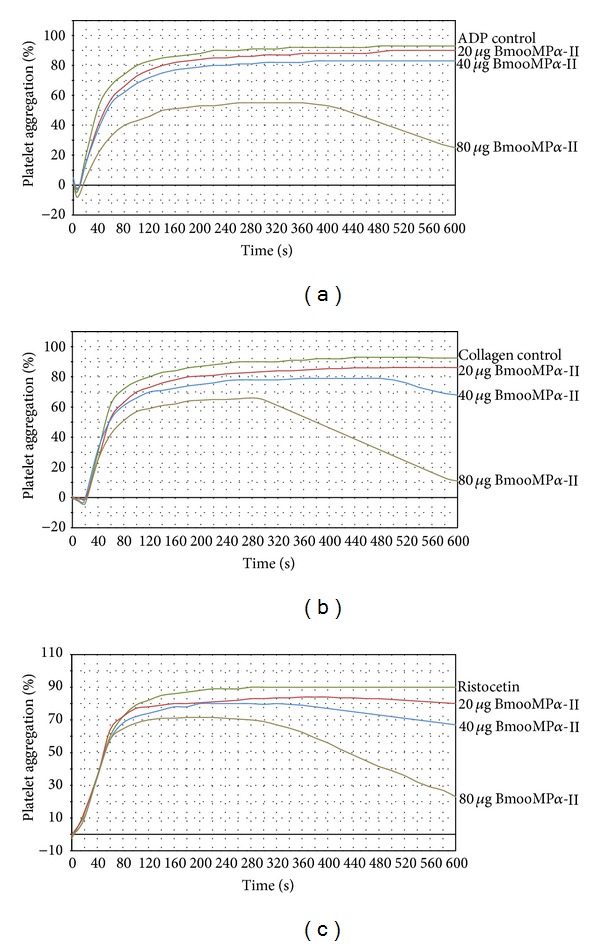
Effect of BmooMP*α*-II (20, 40, and 80 *μ*g) from* B. moojeni* venom on (a) ADP, (b) collagen, and (c) ristocetin-induced platelet aggregation. Aggregation was triggered with agonists immediately after adding the indicated doses of BmooMP*α*-II to human PRP at 37°C. Platelet aggregation was recorded for 10 min in an AggRAM platelet aggregation system with four-channel laser optics (Helena Laboratories, EUA). Results were expressed as an increase in light transmission, where PPP represents the maximum response (100%). Control experiments were performed in the absence of BmooMP*α*-II.

BmooMP*α*-II belongs to PI classes of metalloproteinases and therefore it is devoid of disintegrin or disintegrin-like domains. Interestingly, BmooMP*α*-II inhibited collagen, ADP, and ristocetin-induced platelet aggregation in a concentration-dependent manner. Our results showed that 80 *μ*g of BmooMP*α*-II inhibited over 80% of agonists-induced platelet aggregation (Figure 8). The absence of desintegrin and desintegrin-like domains suggests that the enzyme does not cause inhibition of platelet aggregation via interaction with membrane receptors. Furthermore, inhibition of the platelet aggregation caused by BmooMP*α*-II appears to begin after some time of start of the assay. These results suggest that BmooMP*α*-II inhibits platelet aggregation due to hydrolysis of the integrin *α*_IIb_ *β*_3_. This receptor plays a central role in linking activated platelets. Independent of the initial stimulus, blocking or hydrolysis of integrin *α*_IIb_ *β*_3_ prevents platelet aggregation and subsequent thrombus formation by preventing binding to fibrinogen. The participation of integrin *α*_IIb_ *β*_3_ in platelet aggregation, whatever the initiating event or agonist, justifies the interest in therapeutic blockade of this receptor, since all routes of platelet activation converge on to this final common pathway [18, 50, 51].

BmooMP*α*-II degrades bovine fibrinogen, the major ligand for the platelet *α*_IIb_ *β*_3_ integrin. However, the hydrolysis of A*α* and B*β* chains of fibrinogen by BmooMP*α*-II is not responsible for the inhibition of platelet aggregation since it does not hydrolyze the *γ* chain, which contains the more important platelet-binding site [52].

## 4. Conclusion

In conclusion, our results suggest that BmooMP*α*-II from* B. moojeni* venom belongs to class P-I of SVMPs and probably is an *α*-fibrinogenase. BmooMP*α*-II induced relevant histological changes in the liver, lungs, kidneys, and muscle from mice, contributing to the toxic effect of the envenomation caused by* B. moojeni* venom. Moreover, the enzymatically active protein inhibited collagen, ADP, and ristocetin-induced platelet aggregation in a concentration-dependent manner. Anyway, more investigations about BmooMP*α*-II are needed to elucidate the mechanisms of inhibition and may contribute to the basic studies of* B. moojeni* venom of platelet function and for the development of novel therapeutic agents to prevent and treat thrombotic disorders.

## References

[1] Calgarotto AK, Damico DCS, Ponce-Soto LA (2008). Biological and biochemical characterization of new basic phospholipase A_2_ BmTX-I isolated from *Bothrops moojeni* snake venom. *Toxicon*.

[2] Markland FS (1998). Snake venoms and the hemostatic system. *Toxicon*.

[3] Ramos OHP, Selistre-De-Araujo HS (2006). Snake venom metalloproteases—structure and function of catalytic and disintegrin domains. *Comparative Biochemistry and Physiology C: Toxicology & Pharmacology*.

[4] Chippaux J-P, Williams V, White J (1991). Snake venom variability: methods of study, results and interpretation. *Toxicon*.

[5] Calvete JJ, Marcinkiewicz C, Monleón D (2005). Snake venom disintegrins: evolution of structure and function. *Toxicon*.

[6] Costa JDO, Fonseca KC, Mamede CCN (2010). Bhalternin: functional and structural characterization of a new thrombin-like enzyme from *Bothrops alternatus* snake venom. *Toxicon*.

[7] Della-Casa MS, Junqueira-de-Azevedo I, Butera D (2011). Insularin, a disintegrin from *Bothrops insularis* venom: inhibition of platelet aggregation and endothelial cell adhesion by the native and recombinant GST-insularin proteins. *Toxicon*.

[8] Denegri MEG, Acosta OC, Huancahuire-Vega S (2010). Isolation and functional characterization of a new acidic PLA_2_ Ba SpII RP4 of the *Bothrops alternatus* snake venom from Argentina. *Toxicon*.

[9] Gomes MSR, Mendes MM, de Oliveira F (2009). BthMP: a new weakly hemorrhagic metalloproteinase from *Bothrops moojeni* snake venom. *Toxicon*.

[10] Gomes MSR, de Queiroz MR, Mamede CCN (2011). Purification and functional characterization of a new metalloproteinase (BleucMP) from *Bothrops leucurus* snake venom. *Comparative Biochemistry and Physiology C: Toxicology & Pharmacology*.

[11] Singletary EM, Rochman AS, Bodmer JCA, Holstege CP (2005). Envenomations. *Medical Clinics of North America*.

[12] Queiroz MR, Mamede CC, Fonseca KC (2011). Biological characterization of a myotoxin phosphoplipase A_2_ homologue purified from the venom of the snake **Bothrops moojeni**. *Journal of Venomous Animals and Toxins Including Tropical Diseases*.

[13] Sajevic T, Leonardi A, Križaj I (2011). Haemostatically active proteins in snake venoms. *Toxicon*.

[14] Santos-Filho NA, Silveira LB, Oliveira CZ (2008). A new acidic myotoxic, anti-platelet and prostaglandin I2 inductor phospholipase A_2_ isolated from *Bothrops moojeni* snake venom. *Toxicon*.

[15] Gutiérrez JM, Rucavado A (2000). Snake venom metalloproteinases: their role in the pathogenesis of local tissue damage. *Biochimie*.

[16] Gutiérrez JM, Rucavado A, Escalante T, Díaz C (2005). Hemorrhage induced by snake venom metalloproteinases: biochemical and biophysical mechanisms involved in microvessel damage. *Toxicon*.

[17] Rodrigues VM, Soares AM, Andrião-Escarso SH (2001). Pathological alterations induced by neuwiedase, a metalloproteinase isolated from *Bothrops neuwiedi* snake venom. *Biochimie*.

[18] Kamiguti AS (2005). Platelets as targets of snake venom metalloproteinases. *Toxicon*.

[19] Ministério da Saúde (2001). *Manual de Diagnóstico e Tratamento de Acidentes por Animais Peçonhentos*.

[20] Nogueira C, Sawaya RJ, Martins M (2003). Ecology of the Pitviper, *Bothrops moojeni*, in the Brazilian Cerrado. *Journal of Herpetology*.

[21] Itzhaki RF, Gill DM (1964). A micro-biuret method for estimating proteins. *Analytical Biochemistry*.

[22] Laemmli UK (1970). Cleavage of structural proteins during the assembly of the head of bacteriophage T4. *Nature*.

[23] Rodrigues VM, Soares AM, Guerra-Sá R, Rodrigues V, Fontes MRM, Giglio JR (2000). Structural and functional characterization of neuwiedase, a nonhemorrhagic fibrin(ogen)olytic metalloprotease from Bothrops neuwiedi snake venom. *Archives of Biochemistry and Biophysics*.

[24] Nikai T, Mori N, Kishida M, Sugihara H, Tu AT (1984). Isolation and biochemical characterization of hemorrhagic toxin f from the venom of *Crotalus atrox* (western diamondback rattlesnake). *Archives of Biochemistry and Biophysics*.

[25] Gene JA, Roy A, Rojas G, Gutierrez JM, Cerdas L (1989). Comparative study on coagulant, defibrinating, fibrinolytic and fibrinogenolytic activities of Costa Rican crotaline snal venoms and their neutralization by a polyvalent antivenom. *Toxicon*.

[26] Marcussi S, Bernardes CP, Santos-Filho NA (2007). Molecular and functional characterization of a new non-hemorrhagic metalloprotease from *Bothrops jararacussu* snake venom with antiplatelet activity. *Peptides*.

[27] Bernardes CP, Santos-Filho NA, Costa TR (2008). Isolation and structural characterization of a new fibrin(ogen)olytic metalloproteinase from *Bothrops moojeni* snake venom. *Toxicon*.

[28] Sanchez EF, Schneider FS, Yarleque A (2010). The novel metalloproteinase atroxlysin-I from Peruvian *Bothrops atrox* (Jergón) snake venom acts both on blood vessel ECM and platelets. *Archives of Biochemistry and Biophysics*.

[29] Stroka A, Donato JL, Bon C, Hyslop S, de Araújo AL (2005). Purification and characterization of a hemorrhagic metalloproteinase from *Bothrops lanceolatus* (Fer-de-lance) snake venom. *Toxicon*.

[30] Costa FLS, Rodrigues RS, Izidoro LFM (2009). Biochemical and functional properties of a thrombin-like enzyme isolated from *Bothrops pauloensis* snake venom. *Toxicon*.

[31] Magalhães A, Magalhães HPB, Richardson M (2007). Purification and properties of a coagulant thrombin-like enzyme from the venom of *Bothrops leucurus*. *Comparative Biochemistry and Physiology A: Molecular and Integrative Physiology*.

[32] Bello CA, Hermogenes ALN, Magalhaes A (2006). Isolation and biochemical characterization of a fibrinolytic proteinase from *Bothrops leucurus* (white-tailed jararaca) snake venom. *Biochimie*.

[33] Gutiérrez J, Romero M, Díaz C, Borkow G, Ovadia M (1995). Isolation and characterization of a metalloproteinase with weak hemorrhagic activity from the venom of the snake *Bothrops asper* (terciopelo). *Toxicon*.

[34] Rucavado A, Núñez J, Gutiérrez JM (1998). Blister formation and skin damage induced by BaP1, a haemorrhagic metalloproteinase from the venom of the snake *Bothrops asper*. *International Journal of Experimental Pathology*.

[35] Watanabe L, Shannon JD, Valente RH (2003). Amino acid sequence and crystal structure of BaP1, a metalloproteinase from *Bothrops asper* snake venom that exerts multiple tissue-damaging activities. *Protein Science*.

[36] Patiño AC, Pereañez JA, Núñez V (2010). Isolation and biological characterization of Batx-I, a weak hemorrhagic and fibrinogenolytic PI metalloproteinase from Colombian *Bothrops atrox* venom. *Toxicon*.

[37] Baldo C, Tanjoni I, León IR (2008). BnP1, a novel P-I metalloproteinase from *Bothrops neuwiedi* venom: biological effects benchmarking relatively to jararhagin, a P-III SVMP. *Toxicon*.

[38] Okamoto DN, Kondo MY, Oliveira LCG (2014). P-I class metalloproteinase from *Bothrops moojeni* venom is a post-proline cleaving peptidase with kininogenase activity: insights into substrate selectivity and kinetic behavior. *Biochimica et Biophysica Acta*.

[39] Costa JDO, Fonseca KC, Garrote-Filho MS (2010). Structural and functional comparison of proteolytic enzymes from plant latex and snake venoms. *Biochimie*.

[40] Boer-Lima PA, Gontijo JAR, da Cruz-Höfling MA (1999). Histologic and functional renal alterations caused by *Bothrops moojeni* snake venom in rats. *American Journal of Tropical Medicine and Hygiene*.

[41] Zychar BC, Dale CS, Demarchi DS, Gonçalves LRC (2010). Contribution of metalloproteases, serine proteases and phospholipases A_2_ to the inflammatory reaction induced by *Bothrops jararaca* crude venom in mice. *Toxicon*.

[42] Soares AM, Marcussi S, Stábeli RG (2003). Structural and functional analysis of BmjMIP, a phospholipase A_2_ myotoxin inhibitor protein from *Bothrops moojeni* snake plasma. *Biochemical and Biophysical Research Communications*.

[43] Matsui T, Fujimura Y, Titani K (2000). Snake venom proteases affecting hemostasis and thrombosis. *Biochimica et Biophysica Acta*.

[44] Zhou Q, Dangelmaier C, Smith JB (1996). The hemorrhagin catrocollastatin inhibits collagen-induced platelet aggregation by binding to collagen via its disintegrin-like domain. *Biochemical and Biophysical Research Communications*.

[45] Chen R-Q, Jin Y, Wu J-B (2003). A new protein structure of P-II class snake venom metalloproteinases: it comprises metalloproteinase and disintegrin domains. *Biochemical and Biophysical Research Communications*.

[46] Jangprasert P, Rojnuckarin P (2014). Molecular cloning, expression and characterization of albolamin: a type P-IIa snake venom metalloproteinase from green pit viper (*Cryptelytrops albolabris*). *Toxicon*.

[47] Kamiguti AS, Hay CRM, Theakston RDG, Zuzel M (1996). Insights into the mechanism of haemorrhage caused by snake venom metalloproteinases. *Toxicon*.

[48] Song J, Xu X, Zhang Y (2013). Purification and characterization of AHPM, a novel non-hemorrhagic P-IIIc metalloproteinase with *α*-fibrinogenolytic and platelet aggregation-inhibition activities, from *Agkistrodon halys pallas* venom. *Biochimie*.

[49] Wang W-J (2007). Purification and functional characterization of AAV1, a novel P-III metalloproteinase, from Formosan *Agkistrodon acutus* venom. *Biochimie*.

[50] Shlansky-Goldberg R (2002). Platelet aggregation inhibitors for use in peripheral vascular interventions: what can we learn from the experience in the coronary arteries?. *Journal of Vascular and Interventional Radiology*.

[51] Speich HE, Earhart AD, Hill SN (2009). Variability of platelet aggregate dispersal with glycoprotein IIb-IIIa antagonists eptifibatide and abciximab. *Journal of Thrombosis and Haemostasis*.

[52] Kamiguti AS, Zuzel M, Theakston RDG (1998). Snake venom metalloproteinases and disintegrins: interactions with cells. *Brazilian Journal of Medical and Biological Research*.

